# P-1847. Predictors of Adverse Drug Events and Readmissions Prior to Programmatic Strengthening of an Outpatient Parenteral Antimicrobial Therapy (OPAT) Program

**DOI:** 10.1093/ofid/ofaf695.2016

**Published:** 2026-01-11

**Authors:** Neha Nanda, Austin Fan, Melissa Krainson

**Affiliations:** USC, los angeles, CA; Keck School of Medicine of the University of Southern California, Los Angeles, CA; University of Southern California, Division of Infectious Diseases, Los Angeles, California

## Abstract

**Background:**

OPAT allows for decreased length of hospital stay in patients who require intravenous antimicrobial therapy, but upon discharge, they are at high risk of adverse drug events (ADE) and readmission. We assessed the prevalence and risk factors for ADEs for patients enrolled in OPAT prior to programmatic strengthening. Current proposed changes include a structured ID physician and physician associate led OPAT program to help facilitate the complex care coordination needed between hospitals, outpatient clinics, home health agencies, and the patient.

**Methods:**

Adult patients discharged from an academic hospital with OPAT from October 2024 - February 2025 were included in this retrospective study. Data on patient characteristics and outcomes were collected from electronic medical records for 90 days after enrollment. Multivariate analysis was conducted to identify predictors of ADEs and readmissions.

**Results:**

In total, 78 patients were enrolled in the OPAT program. Of the 78 patients, 73 (93.5%) were discharged on IV antibiotics and were further analyzed to understand our OPAT population. Of the 73 patients, more than half the patients had missing/delayed OPAT labs (52%, 38/73). Among the patients with missing labs, 57.9% (22/38) were discharged home with home health services and the remainder were either discharged to a skill nursing facility (10/38, 26.3%) or were transferred back to the original hospital (6/38,15.8%).

Among the 73 patients, 21 (28.8%) experienced an ADE of which 10 (13.7%) experienced a severe/serious ADE. Multi-drug OPAT receipt (OR: 1.25; 95% CI: 1.06-1.39); p< 0.01) was associated with increased odds of severe/serious ADE. Twenty-two (22/73; 30.1%) patients experienced an unplanned 90-day OPAT-related readmissions, which included infection recurrence or progression and ADE. Missing/delayed OPAT labs (OR: 1.4; 95% CI: 1.1-1.58); p< 0.01) was associated with an increased odd of OPAT-related readmission. Location of discharge did not predict likelihood of ADE or readmission.

**Conclusion:**

Adverse drug events and readmissions occurred frequently in our cohort. Future studies are required to assess if a structured OPAT program may reduce rates of ADEs and OPAT-related readmissions.

**Disclosures:**

All Authors: No reported disclosures

